# Ethylene-Mediated VvERF003 Promotes Flavonol Accumulation by Upregulating *VvFLS1* and *VvCHI1* in ‘Chardonnay’ Grape Berry Skin

**DOI:** 10.3390/biom16010069

**Published:** 2026-01-01

**Authors:** Jinjun Liang, Meijie Wang, Yijun Wu, Chongxin Yang, Hui Shang, Pengfei Zhang, Pengfei Wen

**Affiliations:** College of Horticulture, Shanxi Agricultural University, Jinzhong 030801, China; liangjinjun@sxau.edu.cn (J.L.); z20223287@stu.sxau.edu.cn (M.W.); 20232266@stu.sxau.edu.cn (Y.W.); 20233327@stu.sxau.edu.cn (C.Y.); 20233316@stu.sxau.edu.cn (H.S.); zpflyl@sxau.edu.cn (P.Z.)

**Keywords:** grape, flavonols biosynthesis, ethylene, 1-MCP, *VvERF003*

## Abstract

Flavonols are an important secondary metabolite in grape, which play a crucial role in plant growth and development, human health, and wine making. Ethylene and its inhibitor 1-Methylcyclopropene (1-MCP) are widely used in grape berry production. However, the regulation mechanism of flavonol biosynthesis by ethylene and 1-MCP remains elusive in yellow-green grape varieties. Here, the content of flavonols in ‘Chardonnay’ grape berry skin after ethylene treatment was significantly higher than the control, while 1-MCP treatment was lower than the control. The phenylpropanoid biosynthesis-related genes and a transcription factor *VvERF003* were screened for possible involvement in ethylene-mediated flavonol biosynthesis by transcriptome sequencing. The role of *VvERF003* was further proved to promote flavonol accumulation in the transient overexpression of grape fruits and leaves, and the upregulation of genes related to flavonol biosynthesis. Furthermore, *VvERF003* promoted flavonol biosynthesis by directly binding to and activating the promoters of *VvCHI1* and *VvFLS1*, and positively regulated their expression. These results indicated that *VvERF003* was induced by ethylene and promoted the accumulation of flavonols in ‘Chardonnay’ grape berry skin by positively regulating the flavonol biosynthesis genes *VvCHI1* and *VvFLS1*.

## 1. Introduction

Flavonols, a common flavonoid compound with a C6-C3-C6 basic skeleton, typically exist in fruits in the form of glycosides [1]. They not only play an important role in plant growth, development, and stress resistance [2,3,4], but also play a crucial role in preventing tumors, cardiovascular disease, hyperglycemia, and hyperlipidemia in humans due to their strong antioxidant activity [5,6,7]. Furthermore, flavonols have an impact on the sensory perception of bitterness and astringency in wine, while also influencing color stability [8]. The biosynthetic pathway of flavonols has been identified in plants [9]. Phenylalanine is catalyzed by phenylalanine ammonia lyase (PAL), cinnamic 4-hydroxylase (C4H), and 4-coumarate: CoA ligase (4CL) to form p-Coumaroyl-CoA, which constitutes the fundamental phenylpropane pathway. Subsequently, naringin is generated via the activity of chalcone synthase (CHS) and chalcone isomerase (CHI). Naringin undergoes further hydroxylation through the action of flavonoid 3-hydroxylase (F3H), flavonoid 3’-hydroxylase (F3′H), or flavonoid 3′,5′-hydroxylase (F3′5′H) to produce various dihydroflavonol compounds [1]. Dihydroflavonol is oxidized by flavonol synthase (FLS) to form flavonol [10].

Ethylene (ETH) is an important plant growth regulator in the process of plant growth and development. It binds to the ethylene receptor protein ETRs, causing inactivation of the ETR-CTR1 complex and activation of EIN2 [11]. The ethylene signal downstream of *EIN2* is mediated by the EIN3 gene family, including *EIN3* and *EIN3 like 1* (*EIL1*), which plays a critical role in the ethylene signaling pathway [12]. Ethylene response factors (ERFs) are an important class of transcription factors that participate in plant responses to ethylene signals and play key roles in plant growth, development, and stress resistance. The ERF transcription factor family is induced by EIN3, which subsequently activates or inhibits the expression of downstream genes. 1-MCP, as an ethylene antagonist, competitively binds to ethylene receptors to inhibit subsequent ethylene reactions. For instance, ethylene and 1-MCP treatments increased and decreased the lignin content in kiwifruit, respectively, through regulating many phenylpropane pathway genes such as PAL, C4H, and CHS [13]. ERF transcription factors are widely involved in the phenylpropanoid metabolism pathway in plants. In red-fleshed pears, *PcERF5* interacts with *PcMYB10* to create a complex, which subsequently stimulated the expression of flavonoid synthesis pathway genes such as *PcDFR*, *PcANS*, and *PcUFGT*, ultimately elevating the anthocyanin content in the pear flesh [14].

Overexpression of *CsERF003* in citrus fruits increased the content of the main precursor phenylalanine flavonoids, and upregulated the expression of key genes (*CHS* and *CHI*) involved in the initiation of flavonoid biosynthesis in the phenylpropanoid metabolic pathways (*PAL*, *4CH*, *4CL*) [15]. In tomatoes, overexpression of *SlERF.G3-like* activated the expression of *SlFLS* and other early genes in the flavonoid biosynthesis pathway, such as *SlCHS1/2*, *SlCHI*, *SlF3H*, and *SlF3’H*, thereby inducing an increase in flavonoid content in the fruit [16].

Grapes are a non-climacteric fruit, but ethylene also plays an important role in the growth and development of grape fruits. Ethylene treatment could promote the accumulation of anthocyanins in ‘Cabernet Sauvignon’ grapes under light conditions [17]. Wang et al. (2022) [18] found that ethylene improved fruit color by increasing the expression of anthocyanin-related genes and decreasing the expression of chlorophyll-related genes. In the red grape variety ‘Merlot’, melatonin triggers the expression of *VvMYB14*, which enhances ethylene production by activating the transcription of *VvACS1*, ultimately influencing the buildup of secondary metabolites in the berry skin, such as flavonoids, phenolic acids, stilbene, and flavonols [19]. In addition, ethylene treatment has the potential to influence the color transition of grape berry skin by decreasing the chlorophyll content [20]. Although many studies have examined the impact of exogenous ethylene on the synthesis or breakdown of secondary metabolites in grapes, its regulation of flavonol biosynthesis in yellow-green grapes, such as ‘Chardonnay’, remains unreported.

Here, we identified an ERF transcription factor *VvERF003* regulated by exogenous ethylene through transcriptome sequencing, and explored its regulatory mechanism on the biosynthesis of flavonols in the yellow-green variety ‘Chardonnay’ grape berry skin. The regulatory mechanism of plant growth regulators on the biosynthesis of flavonols in grape berry skin has been further improved, providing a theoretical foundation for the exogenous application of ethylene in regulating grape growth and development in practical production.

## 2. Materials and Methods

### 2.1. Plant Material

The grape variety ‘Chardonnay’ was cultivated in the wine grape harvesting garden of the Institute of Fruit Trees of Shanxi Agricultural University, and was routinely cultivated and managed. The veraison fruits were treated with ethephon (500 mg/L) and 1-MCP (1 μL/L), and water was sprayed as a control. After the treatment, berry skin samples were collected at 0 h, 6 h, 12 h, 24 h, 48 h, 72 h, 5 d, 10 d, 15 d, 20 d, and 25 d, respectively, and the berry skin was separated immediately after sampling, and the liquid nitrogen was frozen and stored at −80 °C. Samples taken within 72 h after treatment were utilized for gene expression analysis, while samples collected between 5 and 25 days post-treatment were employed for the determination of physiological and biochemical indicators. Tobaccos were cultivated in an artificial climate chamber (Ningbo Ledian Instrument Manufacturing Co., Ltd., Ningbo, China) maintained at 25 °C with a light intensity of 0.91 μmol m^−2^ s^−1^, and subjected to a 16 h light/8 h dark cycle.

### 2.2. Determination of Total Flavonol Content

The total flavonol contents were determined by referring to the instructions provided by Shanghai Enzyme-Linked Biologics in Shanghai, China. For each sample, three biological replicates were set up.

### 2.3. RNA Extraction and Transcriptome Sequencing

Total RNA was extracted by the CTAB method, and grape berry skin RNA was extracted at 0 h and 24 h after treatment with ethephon and 1-MCP, followed by transcriptome sequencing. Quality control, library construction, and sequencing were performed by Bioengineering Co., Ltd, Shanghai, China. using the Illumina HiSeq^TM^ platform. The raw data of sequencing were evaluated for quality by FastQC. DESeq was used for differentially expressed gene (DEG) analysis, and a DEG with qValue < 0.05 and difference multiple |FoldChange| > 2 was screened as significantly expressed differential genes. The DAVID database was used to annotate the GO function and analyze the enrichment of the KEGG pathway for the DEGs.

### 2.4. qRT-PCR

Total RNA was reverse transcribed into cDNA by using a reverse transcription kit (Yugong Biologics, Lianyungang, China). The gene CDS sequence was downloaded from the Ensemble database and qRT-PCR primers were designed using Primer Premier 5 software (Supplementary Table S1). *VvUBQ* was used as the internal reference gene, and the PCR reaction was carried out using the QuantStudio^TM^ 3 real-time PCR instrument (Juhemei, Beijing, China). Three biological replicates were set, and the relative expression of genes was calculated by the 2^−△△Ct^ method.

### 2.5. Subcellular Localization

According to the CDS sequence of *VvERF003* with terminators removed, a GFP fusion expression vector was constructed and transformed into agrobacterium GV3101. Agrobacterium solution transformed into fusion expression vector (35S::VvERF003-GFP) and empty carrier (35S::GFP) were injected into tobacco leaves, and fluorescence was observed by laser confocal microscopy (Leica, Wetzlar, Germany) after two days of incubation.

### 2.6. Transient Overexpression

The CDS sequence of *VvERF003* was inserted into the pCAMBIA-35S-1300 vector, and the 35S::VvERF003 overexpression fusion vector was constructed; the empty vector was used as the negative control. The overexpression vector and no-load were transformed into agrobacterium GV3101 (Coolaber, Beijing, China) by the freeze–thaw method. About 5cm of ‘Chardonnay’ grape leaves was taken, the transformed agrobacterium solution was used for vacuum osmosis infection, and the ‘Chardonnay’ grape fruits were injected in the turning color stage. The treated leaves and fruits were cultured in an artificial climate box at 25 °C, 3000 lux in light intensity, and 16 h/8 h alternating light and dark, and sampled at 4 and 6 days after treatment, respectively.

### 2.7. Yeast One-Hybrids

The AD-VvERF003 expression vector was constructed based on the CDS sequence of *VvERF003*, with pGADT7 serving as a negative control. The promoter of the target gene was truncated to a sequence of about 200 bp containing the ERF transcription factor binding site GCC-box (A/GCCGCC) or DRE-motif (G/ACCGAC). Subsequently, decoy vectors such as pAbAi-CHI1, pAbAi-FLS1, pAbAi-CHI2, and pAbAi-F3H2 were also constructed. The negative control vectors pAbAi-chi1, pAbAi-fls1, pAbAi-chi2, and pAbAi-f3h2 with mutations were synthesized by Bioengineering (Shanghai) Co., Ltd. in China (Supplementary Table S1). The AD-VvERF003/pGADT7 +pAbAi-CHI1, pAbAi-FLS1, pAbAi-CHI2, and pAbAi-F3H2 and the AD-VvERF003 + pAbAi-chi1, pAbAi-fls1, pAbAi-chi2, and pAbAi-f3h2 were co-transformed into yeast competent cells, respectively, and coated on SD/-Leu medium plates. After the colonies had grown, they were inoculated into SD/-Leu-AbA medium to observe the growth with varying concentrations of AbA.

### 2.8. Double-Luciferase Experiment

In the promoter sequence of the first 2000 bp of ATG of the VvCHI1 and VvFLS1 gene, the ERF transcription factor binding sites, namely the GCC-box (A/GCCGCC) and DRE-motif (G/ACCGAC), were manually searched. Subsequently, the promoter region containing the ERF binding site was inserted into the upstream of the LUC reporter in the pGreenII0800-LUC vector to generate the pro-LUC reporter vector. The effector vector (35S::VvERF003) was used for overexpression, while the no-load vector (pCAMBIA-35S-1300) served as the negative control. The reporter vector and effector vector were mixed in a 1:1 ratio and injected into tobacco leaves. The transcriptional activation activity of the gene was assessed as LUC/REN, which were quantified with the Dual Luciferase Reporter Assay Kit (Vazyme, Nanjing, China), with three biological replicates per sample.

### 2.9. Data Statistics and Analysis

Data analysis was performed using Microsoft Excel 2010 and GraphPad 8.0. The significant differences between the two groups of data were analyzed by multiple comparisons, with * representing *p* < 0.05, ** representing *p* < 0.01, *** representing *p* < 0.001, and **** representing *p* < 0.0001. Data were expressed as mean ± SD with at least 3 biological replicates per set of data.

## 3. Results

### 3.1. Exogenous Ethylene Promotes the Accumulation of Total Flavonols in Grape Berry Skin

**Figure 1:**
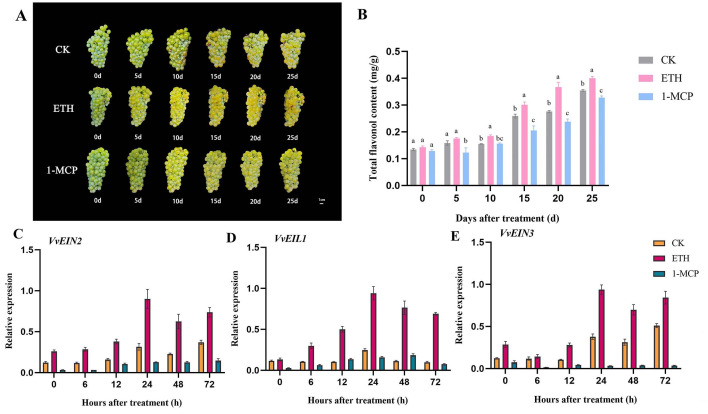
Exogenous ethylene promotes the biosynthesis of total flavonols in grape berry skin. (**A**) Photos of ‘Chardonnay’ grape fruits from 0 d to 25 d after treatment; (**B**) total flavonol contents at 0 d to 25 d after treatment; (**C**–**E**) relative expression levels of key genes *VvEIN2*, *VvEIL1*, and *VvEIN3* involved in the ethylene signal transduction pathway. Different lowercase letters indicate significant differences (*p* < 0.05).

The veraison period fruit of the yellow-green grape variety ‘Chardonnay’ treated with ethylene was advanced by 10 days; the fruit phenotypic observation was based on the images taken from 0 d to 25 d after treatment (Figure 1A). As the fruit grows and develops, the content of total flavonols in the grape berry skin gradually increases. The content of total flavonols in the ethylene treatment was significantly higher than that of the 1-MCP treatment from the 5th day after treatment to maturity; from the 10th day after treatment to maturity, the total flavonol content in the berry skin of the ethylene treatment was significantly higher than that of the water-control ones (Figure 1B). The expression levels of the key genes *VvEIN2*, *VvEIN3*, and *VvEIL1* of the ethylene signal transduction pathway were increased after ethylene treatment, and the overall expression was higher than that of the control and before treatment (0d), while it was the lowest after 1-MCP treatment (Figure 1C–E). Interestingly, the expression levels of all three genes were the highest at 24 h after ethylene treatment (Figure 1C–E). The results indicated that exogenous ethylene treatment facilitated the synthesis of total flavonols in grape berry skin by activating the ethylene signal transduction pathway, with the peak ethylene signal observed 24 h post-treatment.

### 3.2. Transcriptome Data Quality Analysis

**Figure 2:**
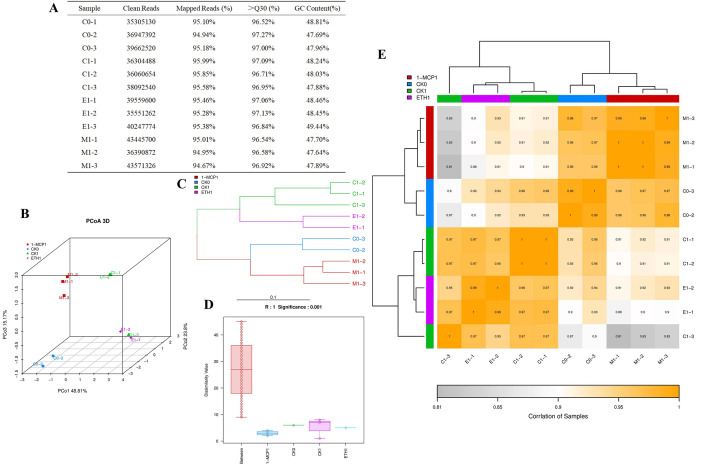
Analysis of transcriptome sample data quality. (**A**) Transcriptome sample data quality; (**B**) principal coordinate analysis (PCoA) plot; (**C**) hierarchical clustering diagram; (**D**) anosim inter-group similarity analysis box plot; (**E**) heatmap for inter-sample correlation analysis.

To investigate the variations in gene expression within grape berry skin following exposure to ethylene and 1-MCP treatments, transcriptome sequencing was conducted on samples collected 24 h post-treatment and 0 h prior to treatment in three distinct groups. A total of 41.31GB of raw data (rowdata) was obtained. After removing the spliced and low-quality sequences, at least 94.67% of the sequences of each sample can be aligned to the grape reference genome. The clean reads of each sample ranged from 3.53 × 10^7^ to 4.35 × 10^7^, the probability of correct recognition of Q30 ranged from 96.52% to 97.27%, and the number of GC bases in the sequence ranged from 47.69% to 48.81% (Figure 2A). The three repeated samples in the group were clustered together, but the groups were relatively scattered using principal coordinate analysis (PCoA) (Figure 2B), indicating that the differences in sequenced biological repeated samples were small, but the differences between different treatments were obvious. The tree branches of each group of samples were clustered together, and the ethylene and 1-MCP branches were far away, suggesting that the processing effects of the two groups were quite different (Figure 2C). The box on the left of the anosim group similarity analysis box chart was longer, indicating that the difference between groups was obvious, while the other boxes were shorter, indicating that the difference within each group was small (Figure 2D). The sample correlation heatmap (Figure 2E) showed that the correlation index of gene expression in each group was greater than 0.99, indicating that the similarity in gene expression patterns between repeated samples was high. The transcriptome sequencing data presented above demonstrated reliability, evident differences between groups, and good intra-group biological repeatability, making it an appropriate sample for analysis.

### 3.3. Ethylene and 1-MCP Treatments Affected the Phenylpropane Biosynthesis Pathway in Grape Berry Skin

**Figure 3:**
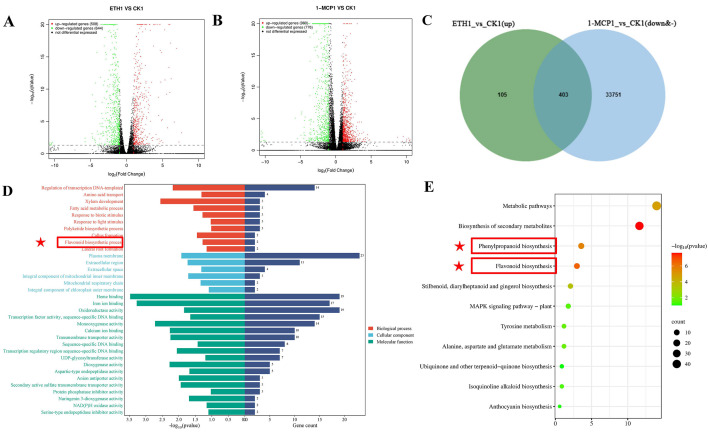
Differential gene screening affecting flavonol biosynthesis. (**A**) Volcano plot of ETH1 vs. CK1 expression differences; (**B**) volcano plot of 1-MCP1 vs. CK1 expression differences; (**C**) Venn diagram of ETH1 vs. CK1 (up) and 1-MCP1 vs. CK1(down&-) co-expression; (**D**) GO enrichment analysis; (**E**) KEGG enrichment analysis. Red star marks pathway related to flavonols.

There were 35,134 differentially expressed genes in the ETH1 vs. CK1 comparison group, of which 508 were upregulated, 644 were downregulated, and 33,982 were not significantly different (Figure 3A). In the 1-MCP1 and CK1 comparison groups, 980 differentially expressed genes were upregulated and 776 differentially expressed genes were downregulated, among which 33,378 differentially expressed genes showed no significant difference (Figure 3B). Taking the intersection of the differentially expressed genes that were significantly upregulated in the ETH1 vs. CK1 comparison group and the differentially expressed genes that were significantly downregulated or not significantly different in the 1-MCP1 vs. CK1 comparison group, 403 genes were found in total (Figure 2C). The 403 genes were annotated into 34 GO entries using GO enrichment analysis (Figure 2D). During the biological process (BP), they were primarily annotated with terms such as “regulation of transcription DNA template”, “amino acid transport”, “xylem development”, “flavoid biosynthetic process”, etc. In terms of molecular function (MF), it mainly noted “heme binding”, “iron binding”, “oxidoreductase activity”, “transcription factor activity, sequence specific DNA binding”, etc. The entries enriched in cell composition (CC) included “plasma membrane”, “extracellular region”, etc. KEGG pathway enrichment analysis showed that the above differentially expressed genes were enriched in 11 pathways, mainly in “metabolic pathways”, “biosynthesis of secondary metabolites”, “phenylpropanoid biosynthesis”, and “flavonoid biosynthesis” (Figure 2E). In conclusion, transcriptome sequencing analysis showed that the differentially expressed genes after ethylene treatment and 1-MCP treatment were significantly enriched in the “phenylpropane biosynthesis pathway”, suggesting a potential association with the biosynthesis of flavonols in grape berry skin.

### 3.4. Screening of Differential Genes Affecting Flavonol Biosynthesis

**Figure 4:**
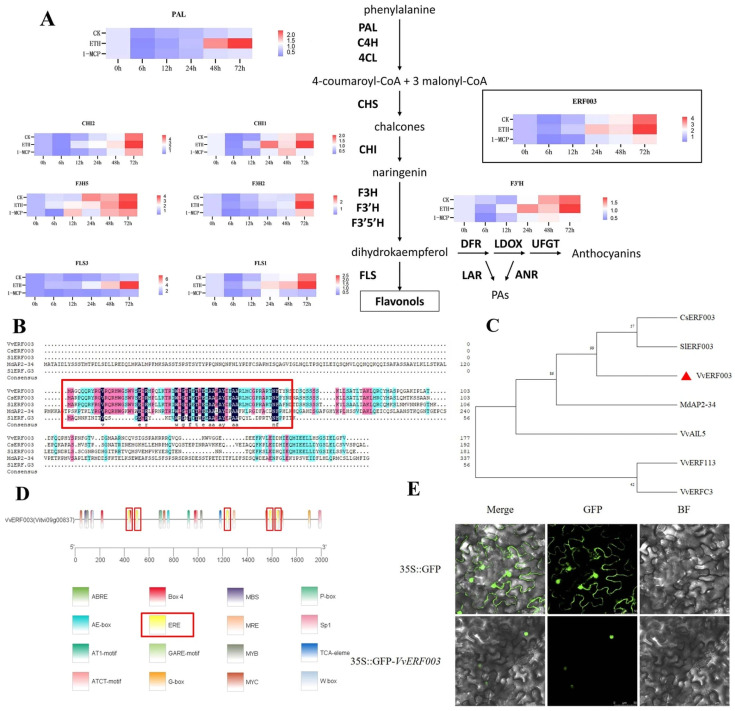
Differential gene screening affecting flavonoid biosynthesis. (**A**) Expression levels of genes related to the flavonoid biosynthesis pathway; (**B**) alignment of conserved domains; (**C**) phylogenetic tree of ERF transcription factors; (**D**) analysis of cis-acting elements in the promoter of *VvERF003*; (**E**) subcellular localization of VvERF003. The red box represents the ethylene responsive elements.

These 403 differentially expressed genes included 8 structural genes of the flavonol biosynthesis pathway. The expression patterns of these genes from 0 h to 72 h in the two treatment groups and the control group were analyzed (Figure 4A). Except for *VvF3H5*, the expression levels of the other seven genes after ethylene treatment were significantly higher than those of the CK and 1-MCP treatment. There was no significant difference in the expression of *VvPAL* and *VvFLS3* between the CK and 1-MCP treatments; the expression of *VvF3H2* was significantly increased at 72 h after ETH treatment, while the expression levels of *VvFLS1* and *VvCHI1* were significantly higher than those of CK and 1-MCP. But in general, the expression of these flavonoid biosynthesis genes showed an upward trend.

Among the above 403 differentially expressed genes, a total of 20 transcription factors were identified, including the MYB family (9), bHLH family (5), AP2/ERF family (4), and NAC family (2) (Supplementary Table S2). Previous research has indicated that ERF003 may participate in phenylpropane metabolism via a specific pathway [15]. Upon comparing the amino acid sequence of *VvERF003* with those of related ERF transcription factors from citrus, tomato, and apple, it was discovered that the conserved domains of *VvERF003* exhibited high levels of similarity with these transcription factors (Figure 4B). The phylogenetic tree comparison between *VvERF003* and the related ERF transcription factors of citrus, tomato, and apple (Figure 4C) showed that *VvERF003*, *CsERF003*, and *SlERF003* were clustered in one branch, indicating high homology, while the other three ERF transcription factors were far from the above genes. These results indicated that *VvERF003* had similar functions with the above citrus and tomato transcription factors, which may affect the biosynthesis of flavonols in grape berry skin. The expression of *VvERF003* was upregulated at 24 h and reached its peak at 72 h after ethylene treatment. Similarly, although the expression was highest at 72 h after water treatment, its overall level was lower compared to that observed in the ethylene treatment group. On the contrary, after 1-MCP treatment, the expression of *VvERF003* generally remained at a low level. The results indicated that *VvERF003* had a similar expression pattern to the structural genes involved in the biosynthesis of flavonols, suggesting that *VvERF003* might regulate the biosynthesis of flavonols. The promoter sequence of *VvERF003* contained five ethylene response elements (Figure 4D), indicating that *VvERF003* may be directly regulated by exogenous ethylene through the ethylene signal transduction pathway. *VvERF003* was located in the nucleus using subcellular localization (Figure 4E). In conclusion, *VvERF003* was potentially involved in the regulation of ethylene-mediated flavonol biosynthesis through regulating its related structural genes.

### 3.5. VvERF003 Promoted the Biosynthesis of Flavonols in Grape Berry Skin

**Figure 5:**
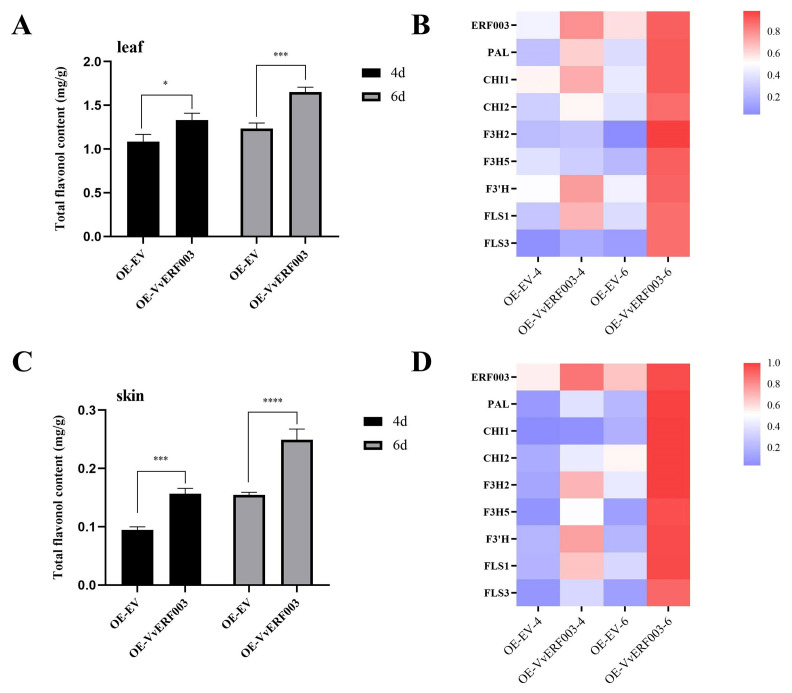
*VvERF003* promoted the biosynthesis of total flavonols in grape berry skin. (**A**) Total flavonol content in transiently overexpressed *VvERF003* and empty vector grape leaves; (**B**) relative expression levels of flavonoid biosynthesis pathway-related genes and *VvERF003* after transient overexpression in grape leaves; (**C**) total flavonol content in grape berry skin after transient overexpression of *VvERF003* and empty vector; (**D**) relative expression levels of flavonoid biosynthesis pathway-related genes and *VvERF003* in grape berry skin after transient overexpression of *VvERF003* and empty vector. The asterisk represents significant difference (*p < 0.05, *** *p* < 0.001, **** *p* < 0.0001).

To verify the impact of *VvERF003* on flavonol biosynthesis in grape berry skin, we overexpressed *VvERF003* in the leaves and fruits of ‘Chardonnay’ grapes. The agrobacterium carrying 35S::VvERF003 and empty vector were infiltrated into grape leaves by vacuum and young fruits by injection, respectively. At 4 and 6 days after infection, the total flavonol contents in leaves and berry skin were significantly higher than those of the control. Four days post-infection, the total flavonol content in leaves overexpressing *VvERF003* was 1.23 times higher than that of the control, while the total flavonol content in berry skin overexpressing *VvERF003* was 1.66 times higher than that of the control (Figure 5A,C). On the sixth day post-infection, the content of total flavonol in the leaves overexpressing *VvERF003* was 1.34 times higher than that of the control, while the content of total flavonol in the berry skin was 1.61 times higher than that of the control (Figure 5A,C). Although the total flavonol content in leaves was higher than that in berry skin, the difference in flavonol content was more obvious after *VvERF003* was overexpressed in berry skin. On the fourth day, the expression levels of *VvF3H2* and *VvF3H5* in leaves were not significantly different from those in the control. In addition, the expression levels of *VvERF003* and structural genes in leaves and berry skins were significantly higher than those in the control on the fourth and sixth days after the overexpression of *VvERF003* (Figure 5B,D). These results indicated that *VvERF003* and key genes of the flavonol biosynthesis pathway were co-expressed to promote the biosynthesis of grape flavonols.

### 3.6. VvERF003 Directly Bound to and Activated the Promoters of VvFLS1 and VvCHI1

**Figure 6:**
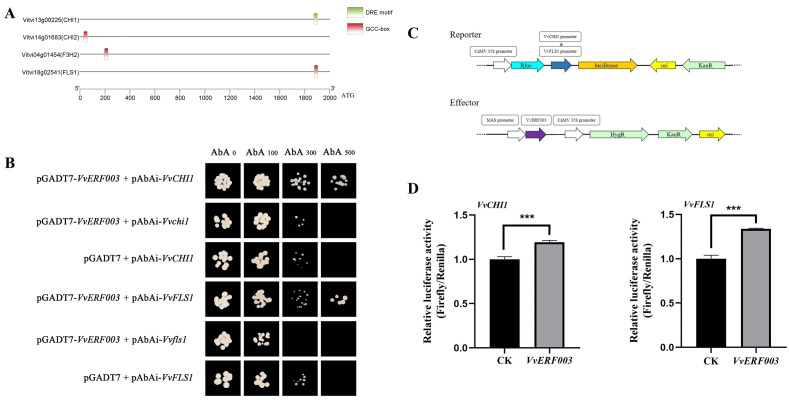
*VvERF003* directly combined to and activated the *VvFLS1* and *VvCHI1* promoters. (**A**) Analysis of cis-acting elements in the promoters of flavonoid biosynthesis pathway-related genes; (**B**) yeast one-hybrid assay to verify the binding of *VvERF003* to the *VvFLS1* and *VvCHI1* promoters, respectively; (**C**) schematic diagram of the dual-luciferase reporter system, including the effector and reporter constructs; (**D**) double-luciferase assay was used to detect the luciferase activity of *VvERF003* co-expressed with the promoters of *VvFLS1* and *VvCHI1*, respectively. The asterisk represents significant difference (*** *p* < 0.001).

In order to further explore how *VvERF003* regulated flavonol biosynthesis, the promoter sequences of the above structural genes were analyzed. Among them, the *VvCHI1* promoter contains a DRE motif, and *VvCHI2*, *VvF3H2*, and *VvFLS1* each contain a GCC box (Figure 6A). To validate this hypothesis, we co-transformed AD-VvERF003 and the vectors carrying the promoters of four structural genes into Yeast One-Hybrid (Y1H) yeast and inoculated it onto a plate containing SD/-Leu-AbA. The results showed that the yeast colonies co-expressing AD-VvERF003, pAbAi-CHI1, and pAbAi-FLS1 could grow on a plate with SD/-Leu-AbA, while the corresponding negative control and empty load were inhibited (Figure 6B). The yeast strains co-expressing AD-VvERF003, pAbAi-CHI2, and pAbAi-F3H2, as well as the corresponding negative control and empty load, were inhibited on a plate with SD/-Leu-AbA (Supplementary Figure S1). These results indicated that *VvERF003* could interact with the promoters of *VvCHI1* and *VvFLS1*, but not with *VvCHI2* and *VvF3H2*. Similarly, the promoters of the VvCHI1 and VvFLS1 structural genes were inserted into the upstream of the firefly luciferase (LUC) gene (Figure 6C) and co-injected into tobacco leaves along with the vector containing the overexpression of *VvERF003*. The results indicated that *VvERF003* notably enhanced the relative luciferase activity driven by the promoters of VvCHI1 (1.19 times) and VvFLS1 (1.34 times) (Figure 6D). In conclusion, VvERF003 can be directly combined with the promoters of *VvCHI1* and *VvFLS1* to activate their expression.

## 4. Discussion

### 4.1. Ethylene and 1-MCP Regulated Flavonol Accumulation in Grape Berry Skin

Ethylene plays an important role in plant growth and development. EIN2 and EIN3/EIL1 were two positive regulators in the ethylene signal transduction pathway [21]. In this study, the expression of *VvEIN2*, *VvEIN3*, and *VvEIL1* was activated by exogenous ethylene, whereas 1-MCP had the opposite effect. This indicated that both exogenous ethylene and 1-MCP exert their influence through the ethylene signal transduction pathway, corroborating the findings of prior research. After ethylene treatment in ‘Kyoho’ grape, the content of chlorophyll (Chl) decreased rapidly with the expression level of *VvERF17*, while the content of Chl in fruits treated with 1-MCP remained basically unchanged, and the expression of *VvERF17* was significantly inhibited [20]. Postharvest 1-MCP treatment led to the accumulation of β-carotenoids in kiwifruit and promoted the degradation of chlorophyll a and b [22]. In banana, 1-MCP significantly delayed fruit maturation by inhibiting fruit softening, reduced the respiration rate and ethylene release, and 1-MCP treatment delayed sugar accumulation and influenced the content of aroma biosynthetic precursors [23]. After treating the red grape variety ‘Rose Fragrance’ with ethylene, 70 DEGs associated with anthocyanin synthesis and accumulation were identified. The majority of these genes were significantly induced by the ethylene treatment. Additionally, during berry ripening, the expression of 16 phenylpropane pathway genes gradually increased and was also strongly induced by the ethylene treatment [18]. In this experiment, the contents of total flavonols in grape berry skin after ethylene treatment were significantly higher than those of the control, whereas they were reduced following 1-MCP treatment. This suggested that ethylene treatment enhances the accumulation of flavonols in grape berry skin, while 1-MCP treatment hinders its accumulation.

### 4.2. VvERF003 Promoted Flavonol Biosynthesis by Directly Binding to VvCHI1 and VvFLS1 Promoters

Our transcriptome sequencing results showed that the related differential genes were significantly enriched in the ‘phenylpropane biosynthesis pathway’, and eight flavonol biosynthesis-related structural genes (*VvPAL*, *VvCHI1*, *VvCHI2*, *VvF3H2*, *VvF3H5*, *VvF3’H*, *VvFLS1*, and *VvFLS3*) and a transcription factor *VvERF003* were found to be responsive to ethylene signaling. Transient overexpression of *CsERF003* in citrus fruits promoted the accumulation of flavonoids and phenylalanine precursors, and the key genes involved in the biosynthesis of phenylpropane (PAL, 4CH, and 4CL) and flavonoids (CHS and CHI) were upregulated after overexpression [15]. In our study, *VvERF003* was transiently overexpressed in grape leaves and fruits to further verify its function and promote flavonol accumulation by upregulating the expression of related genes, which is similar to previous studies conducted on other plants.

**Figure 7:**
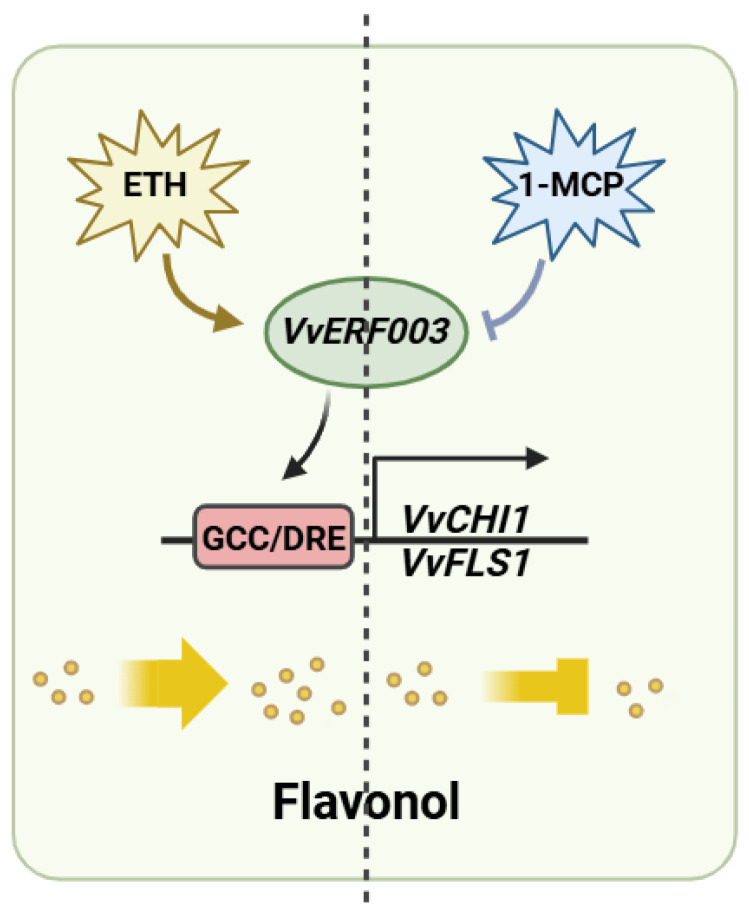
Predicted pattern map of ethylene-mediated regulation of flavonol biosynthesis by *VvERF003*.

ERFs belong to the AP2/ERF superfamily and carry the AP2/ERF domain. The superfamily could be further divided into the AP2 family, ERF family, and RAV family [24]. ERF transcription factors could recognize and bind to the GCC box (A/GCCGCC) and DRE motif (G/ACCGAC) in the downstream gene promoter, thereby activating its expression [7,25]. In grape, *VvERF75* could positively regulate their expression by combining with the DRE motif in the promoters of pheophorbide oxylase (*VvPAO1*) and 1-aminocyclopropane-1-carboxylic acid synthase (*VvACS5*) [26]. Apple *MdAP2-34* activated its expression by directly binding to the DRE motif-acting element on the *MdF3’H* promoter, thereby promoting flavonoid biosynthesis [27]. In this study, the promoters of *VvCHI1* and *VvFLS1* contained a DRE motif and a GCC box, respectively. The Y1H and double-luciferase experiments verified that *VvERF003* could bind to it and activate its expression. In conclusion, our study found that exogenous ethylene promoted the expression of *VvERF003*, contrasting with 1-MCP, which subsequently led to flavonol accumulation by directly binding to and activating the promoters of *VvCHI1* and *VvFLS1* (Figure 7). Additionally, the expression of other flavonol-related structural genes may be modulated by other transcription factor genes following ethylene treatment.

## 5. Conclusions

The content of flavonols in grape berry skin after ethylene treatment was significantly higher than the control, while 1-MCP treatment resulted in lower levels compared to the control. The phenylpropanoid biosynthesis-related genes and a transcription factor *VvERF003* were screened for possible involvement in ethylene-mediated flavonol biosynthesis by transcriptome sequencing. The role of *VvERF003* was further proved to promote flavonol accumulation in the transient overexpression of grape fruits and leaves, and the upregulation of genes related to flavonol biosynthesis. Furthermore, *VvERF003* promoted flavonol biosynthesis by directly binding to and activating the promoters of *VvCHI1* and *VvFLS1*, thereby positively regulating the expression of these two genes. This work indicated that *VvERF003* was induced by ethylene and promoted the accumulation of flavonols in ‘Chardonnay’ grape berry skin by activating the flavonol biosynthesis genes *VvCHI1* and *VvFLS1*.

## Data Availability

The original contributions presented in this study are included in the article/Supplementary Materials. Further inquiries can be directed to the corresponding author.

## Supplementary Materials

The following are available online at https://www.mdpi.com/article/10.3390/biom16010069/s1, Table S1: Primers of this study; Table S2: Differentially expressed transcription factors; Figure S1: Y1H verified the interaction between VvERF003 and the promters of *VvCHI2* and *VvF3H2*.
