# Supplementary material for: Ethylene-Mediated VvERF003 Promotes Flavonol Accumulation by Upregulating VvFLS1 and VvCHI1 in ‘Chardonnay’ Grape Berry Skin
Source: Biomolecules. 2026 Jan 1;16(1):69. doi: 10.3390/biom16010069 (PMC12838987; doi:10.3390/biom16010069)
Supplement: Supplementary file 1 [file biomolecules-16-00069-s001.zip › biomolecules-4026064-supplementary.pdf]

Supplementary Materials

Table S1. Primers of this study.

Function	Gene name	Forward primer sequences (5'-3')	Reverse primer sequences (5'-3')
qRT-PCR	*VvERF003*	CTCGGACCAACTTCACTTACAAT	CGATACTCACCTGGCAATTTCTT
	VvPAL	GGAAACGGGACGGAATCTT	GAGGCAAGCAAGGGGTAATG
	VvCHI1	ATGTCTCCAGTGCCGTCAGTC	CCACAGTCTTGCCCTTCCA
	VvCHI2	AGTTGAATGCTGTCGGCTTGA	CATGTCCTTAGTTGGTTTTGCTG
	VvF3'H	TGAACATCGGGGAGCAAAC	TCGGGCCAGACTCGAATAA
	VvF3H2	AGGCAACCGTGTATCCTCTGA	CAATGGGCTTGGACTCTAACTT
	VvF3H5	ATTATCTGAGCAATGGGAGGTTC	TCTTCAATGCCTTTCGGTTTC
	VvFLS3	AGAGGGGCTCGGTTTGGAA	AGTAGGGTGAGGGCGGACAT
	VvFLS1	CCGCCATTACCATTCTTATCC	GCACCGCCTTGTATTTTCC
	VvEIN3	GTAATCTTGAGTTTATGTCCGTTCC	TCCCATCTTGTGCCCTTGA
	VvEIL1	CCAGTGATTATGATGTTGAGGGTG	TTGTTGTAGGGGCATTGAGTGT
	VvEIN2	GGTGGGTAACTTGCGATGG	GGTGCTGGTTCTTGCTTGTG
Subcellular localization	*VvERF003*	GGGGTACCATGGCTGGACAA	CGCGGATCCCACAAAACCAAG
overexpression	*VvERF003*	GCTCTAGAATGGCTGGACAACAACA	GGACTAGTTTACACAAAACCAAGCTCAAT
Yeast one-hybrid	*VvERF003*	GGAATTCATGGCTGGACAACAAC	*CGCGGATCC*TTACACAAAACCAA
	VvCHI1	CCCAAGCTTTGCCACCAACCTATTG	GGGGTACCATGTGATGTCTGCCTCA
	VvCHI2	CCCAAGCTTTTCCGGTTCACT	GGGGTACCCGTTCAATTCACGA
	VvF3H2	CCCAAGCTTCGACTTCCATTCCTG	GGGGTACCGCTCTTGGATTTCACT
	VvFLS1	*CCCAAGCTT*GCATTAATAAGATCGC	*GGGGTACC*TGAATCACAGGACGA
Dual-luciferase	*VvCHI1*	ACTCACTATAGGGCGAATTGGTACCATCGG TATACACCAGCCTCA	GCGGCCGCTCTAGAACTAGTGGATCCTCGAT GTGATGTCTGCCTCA
	VvFLS1	ACTCACTATAGGGCGAATTGGTACCGGTTG CAAGAGAAGCTAAGTG	GCGGCCGCTCTAGAACTAGTGGATCCATGA ATCACAGGACGAGCAG

Table S2. Differentially expressed transcription factors.

Gene name	Gene id	Mean TPM (ETH1)	Mean TPM (CK1)	log2FoldChange	result	Mean TPM (1-MCP1)	Mean TPM (CK1)	log2FoldChange	result
MYB	Vitvi01g01024	2.1938675	1.074173	1.030250032	up	1.750298333	1.074173	0.704374482	-
	Vitvi19g01742	9.22882	4.572308333	1.013223501	up	6.488289	4.572308333	0.504915386	-
	Vitvi14g01750	9.5530025	1.106576667	3.109850834	up	1.181958667	1.106576667	0.095076177	-
	Vitvi01g00302	3.7838385	0.717581333	2.398636247	up	1.734524	0.717581333	1.273325536	-
	Vitvi15g04655	1.9763025	0.526735333	1.907653644	up	0.947058	0.526735333	0.846374544	-
	Vitvi07g00393	35.672939	13.64545367	1.38640972	up	19.26097	13.64545367	0.49726	-
	Vitvi07g00455	7.1968125	2.942857	1.290140632	up	3.592181667	2.942857	0.287642873	-
	Vitvi07g03055	1.3035185	0.269322667	2.275003498	up	0.356939	0.269322667	0.406341888	-
	Vitvi16g00305	0.4645875	0.0001	12.18173462	up	0.028343	0.0001	8.14684866	-
bHLH	Vitvi08g00719	1.916243	0.0001	14.2259929	up	0.0001	0.0001	0	-
	Vitvi07g02613	0.813087	0.043185	4.234807534	up	0.059280333	0.043185	0.45702327	-
	Vitvi18g00567	0.9856055	0.107282333	3.199597786	up	0.056529667	0.107282333	-0.924332425	-
	Vitvi14g04018	59.766052	18.86576233	1.663555847	up	35.650646	18.86576233	0.918157821	-
	Vitvi01g00232	1.804453	0.854050333	1.079168564	up	0.767647667	0.854050333	-0.1538768	-
AP2/ERF	Vitvi09g00837	20.3449115	8.857395333	1.199713587	up	12.96508967	8.857395333	0.549677766	-
	Vitvi01g01826	6.10923	2.674280667	1.191839671	up	2.15429	2.674280667	-0.311938413	-
	Vitvi11g01231	0.981251	0.219942	2.157499092	up	0.467785333	0.219942	1.088723502	-
	Vitvi05g00715	1.255084	0.326953667	1.940625816	up	0.484963667	0.326953667	0.568790462	-
NAC	Vitvi10g00437	5.873045	2.059422667	1.511868739	up	3.151220333	2.059422667	0.613670679	-
	Vitvi14g01985	30.2874585	14.08996933	1.104052051	up	20.40660133	14.08996933	0.534367454	-

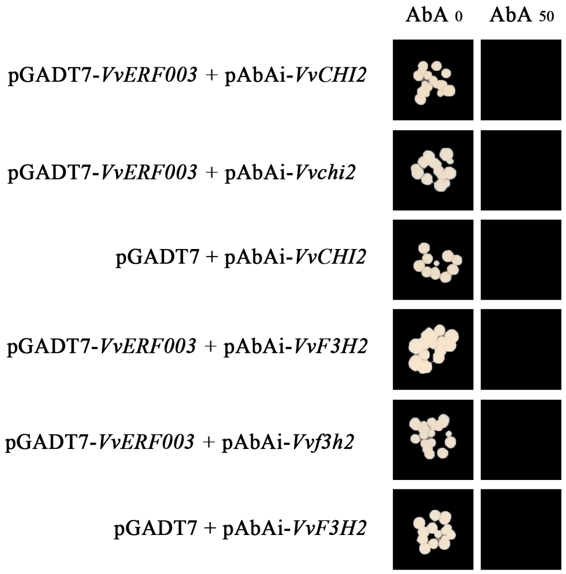

Figure S1. Y1H verified the interaction between VvERF003 and the promters of *VvCHI2* and *VvF3H2*.
